# A Challenging Case of a Large Gastroduodenal Artery Pseudoaneurysm after Surgery of a Peptic Ulcer

**DOI:** 10.1155/2015/370937

**Published:** 2015-01-12

**Authors:** Rocio Santos-Rancaño, Esteban Martín Antona, José Vicente Méndez Montero

**Affiliations:** ^1^Division of Hepatobiliopancreatic Surgery, Department of General Surgery, San Carlos Clinic Hospital of Madrid, Madrid, Spain; ^2^Unit of Abdomen Imaging Diagnosis and Interventional Radiology, Department of Radiology and Imaging, San Carlos Clinic Hospital of Madrid, Madrid, Spain

## Abstract

We report a 48-year-old man in whom a chronic postbulbar duodenal ulcer destroyed much of the back wall of the duodenum and gastroduodenal artery causing pseudoaneurysm. The lesion was found and evaluated by contrast-enhanced computed tomography (that revealed a large pseudoaneurysm of 83 mm × 75 mm in diameter) and by angiography and then treated with transcatheter embolization leading to a complete resolution of the lesion. The case is rare and important for several reasons. First, we demonstrate that pseudoaneurysm of the gastroduodenal artery caused by a duodenal ulcer can occur and present a diagnostic challenge (as far as we know, only three cases have been reported previously in the literature). Second, this case report focuses on the importance of ligation of the gastroduodenal artery when bleeding of peptic ulcers occurs. Additionally, we present an overview of the relevant literature.

## 1. Introduction

Pseudoaneurysms of the gastroduodenal artery are very rare (less than 50 cases reported; 0.01%–0.2% of the autopsies) with the splenic artery being the most common vessel. In addition, their incidence is probably underreported in the literature [1]. They occur as critical complications following pancreatitis and much rarer after gastric or pancreatic surgery or trauma [2]. They are serious because they may be difficult to diagnose and because they may become a life threatening condition if they get ruptured. Therefore, early diagnosis and adequate therapeutic interventions are imperative. At present, the selective embolization of pseudoaneurysms provides a noninvasive tool to manage a disorder that used to be managed by surgery, with a significant reduction of morbimortality.

Herewith we present a challenging case of a 48-year-old man in whom a chronic postbulbar duodenal ulcer eroded gastroduodenal artery causing a giant pseudoaneurysm that was treated with transcatheter embolization leading to a complete resolution of the lesion.

## 2. Case Report

A 48-year-old male patient was admitted to the hospital for hematemesis and melena. His past medical history included chronic alcohol abuse, intense smoking habit, chronic antral gastritis due to* Helicobacter pylori* that had not been eradicated, and longstanding epigastric pain treated with proton pump inhibitors.

The patient was lucid, but anemic, with a fine radial pulse of 120 beats per minute and a blood pressure of 60/40 mm Hg. The abdominal examination showed no elements suggesting peritoneal irritation.

On initial presentation, his hemoglobin level was 7.0 g/dL so the patient management began with the transfusion of two packed red cells and intravenous fluids and posteriorly a gastroscopy was performed revealing a posterior bulbar ulcer of 15 mm, with blood oozing. Hemostasis was achieved using 1/10,000 adrenaline. But the ulcer continued bleeding and, after assuring it was not safe to repeat the sclerosis, we decided to perform an urgent duodenotomy, suture of the penetrated ulcer in the posterior wall and Graham patch. Due to placement of the ulcer and the inflammation of the tissues around, the gastroduodenal artery was not ligated. The patient developed well and was discharged 6 days after. But he presented again to our hospital two days after with a history of persistent epigastric pain associated. He was afebrile and hemodynamically stable; moreover, physical examination revealed a palpable beating mass in the epigastrium.

The contrast-enhanced CT scan documented the presence of a large (8.3 × 7.5 cm) pseudoaneurysm of the gastroduodenal artery supplied by the superior mesenteric artery.

**Figure 1:**
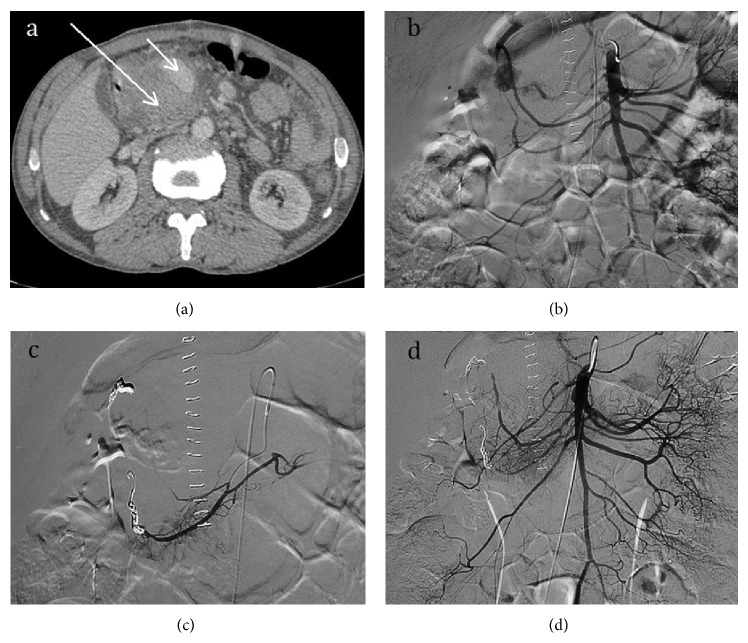
(a) Contrast-enhanced axial CT image shows a giant pseudoaneurysm of 8.3 × 7.5 cm in size originating from the gastroduodenal artery (long arrow). The intravenous contrast showed filling of the mass, certifying its vascular origin (short arrow). (b) Aortography shows the blood circulation of the aneurysm in continuity with the gastroduodenal artery. (c) Angiogram after selective coil-embolization of the gastroduodenal artery through the celiac trunk and the inferior pancreaticoduodenal artery, through the superior mesenteric artery. (d) Angiographic control confirmed the complete exclusion of the pseudoaneurysm.

Selective arterial embolization through a femoral approach was successfully performed to treat the pseudoaneurysm. We decided to occlude the gastroduodenal artery first to stop the backflow into the pseudoaneurysm and it was embolized with two 3 mm × 4 cm coils. Subsequently, the inferior pancreaticoduodenal artery was embolized with two 3 mm × 5 cm coils through the superior mesenteric artery. An angiographic control uncovered a marginal filling of the pseudoaneurysm and an additional embolization using the liquid embolic agent lipiodol/ethibloc mixture was performed. Angiographic control confirmed the complete exclusion of the pseudoaneurysm (Figure 1).

The patient's hospital stay was uneventful and he could be discharged after 4 days without any signs of bleeding or intestinal ischemia. A contrast-enhanced follow-up CT scan (4 weeks after embolization) showed the pseudoaneurysm excluded completely and no changes in its size, which was still thrombosed by the coils.

## 3. Discussion

Pseudoaneurysms represent contained ruptures after injury to one or more layers of a vascular wall. Most of them are a consequence of pancreatitis, much rarer after operative trauma or Whipple procedure, where an enzymatic leak from the pancreaticojejunostomy reconstruction may occasionally lead to breakdown of the ligature on the GDA stump, leading to catastrophic hemorrhage.

In our case a chronic postbulbar duodenal ulcer destroyed much of the back wall of the duodenum, which eroded gastroduodenal artery causing a giant pseudoaneurysm of 8.3 mm × 7.5 mm in diameter. To the best of our knowledge, only three cases have been reported previously in the literature.

The potential for rapid growth and high mortality rates associated with a false aneurysm rupture (up to 100%) emphasizes the importance of early diagnosis and treatment, as in our case [3]. The risk of rupture is not dependent on the size of the aneurysm. Hence, it is advocated that all gastroduodenal artery aneurysms, regardless of size, be treated actively at the time of the diagnosis.

CT is an excellent modality and demonstrates the features of pseudoaneurysm in the majority of cases in the initial differential diagnosis. Angiography also plays a critical role and is considered the gold standard for diagnosis of aneurysms in the peripancreatic vessels [4].

Conservative management of pseudoaneurysms is burdened by a death rate of more than 90%. Endovascular therapies are often favored over surgery, given their less invasive approach, its efficacy rate ranges from 70 to 100% and they have a lower rate of morbimortality. Embolization is done with coils or using percutaneous or endoscopic ultrasound guided thrombin injection [5], as in the present case where the occlusion of the pseudoaneurysm was achieved by filling it with numerous coils and finally with a mixture of ethibloc/lipiodol.

We highlight the importance of this case in several aspects. First, we demonstrate that pseudoaneurysm of the gastroduodenal artery caused by a duodenal ulcer is a possibility with a challenging diagnosis. We also focus on the importance of ligation of the gastroduodenal artery when bleeding of peptic ulcers occurs. Furthermore, our patient did not suffer a serious gastrointestinal bleeding. Additionally, we present an overview of the relevant literature.
